# Mucosal Immune Profiles Associated with Diarrheal Disease Severity in *Shigella*- and Enteropathogenic Escherichia coli-Infected Children Enrolled in the Global Enteric Multicenter Study

**DOI:** 10.1128/mbio.00538-22

**Published:** 2022-08-04

**Authors:** Amanda D. Buskirk, Esther Ndungo, Avital A. Shimanovich, Diana Lam, William C. Blackwelder, Usman N. Ikumapayi, Bing Ma, Helen Powell, Martin Antonio, James P. Nataro, James B. Kaper, Marcela F. Pasetti

**Affiliations:** a Center for Vaccine Development, University of Maryland School of Medicine, Baltimore, Maryland, USA; b Department of Pediatrics, University of Maryland School of Medicine, Baltimore, Maryland, USA; c Department of Microbiology and Immunology, University of Maryland School of Medicine, Baltimore, Maryland, USA; d Institute of Genome Sciences, University of Maryland School of Medicine, Baltimore, Maryland, USA; e Department of Epidemiology and Public Health, University of Maryland School of Medicine, Baltimore, Maryland, USA; f Vaccines and Immunity Theme, Medical Research Council Unit The Gambia, Banjul, The Gambia; g Microbiology and Infection Unit, The University of Warwick, Coventry, United Kingdom; h Faculty of Infectious and Tropical Diseases, London School of Hygiene & Tropical Medicine, London, United Kingdom; i University of Virginia School of Medicine, Department of Pediatrics, Charlottesville, Virginia, USA; University of Michigan—Ann Arbor

**Keywords:** EPEC, GEMS, *Shigella*, mucosal immune profiles

## Abstract

Enteropathogenic Escherichia coli (EPEC) and *Shigella* are etiologic agents of diarrhea in children <5 years old living in resource-poor countries. Repeated bouts of infection lead to lifelong morbidity and even death. The goal of this study was to characterize local mucosal immune responses in *Shigella*- and EPEC-infected children <5 years of age with moderate to severe diarrhea (MSD) enrolled in the Global Enteric Multicenter Study (GEMS). We hypothesized that infection with each of these pathogens would induce distinct gut mucosal immune profiles indicative of disease etiology and severity. To test this hypothesis, innate and adaptive immune markers were measured in stools from children with diarrhea due to EPEC, *Shigella*, or other organisms and in children who had no diarrhea. *Shigella-*positive diarrhea evoked robust proinflammatory and T_H_1/T_H_2 cytokine responses compared to diarrhea caused by EPEC or other organisms, with the exception of interleukin 5 (IL-5), which was associated with EPEC infection. The presence of IL-1β, IL-4, IL-16, and tumor necrosis factor beta (TNF-β) was associated with the absence of dysentery. EPEC-positive diarrhea evoked high levels of IL-1β, vascular endothelial growth factor (VEGF), and IL-10. Granulocyte-macrophage colony-stimulating factor (GM-CSF) had opposing roles in disease severity, being associated with absence of diarrhea in EPEC-infected children and with dysenteric *Shigella* infection. High levels of antigen-specific antibodies were detected in the controls and children with *Shigella* without dysentery, which suggests a protective role against severe disease. In summary, this study identified distinct local immune responses associated with two clinically relevant diarrheagenic pathogens, *Shigella* and EPEC, in children and identified protective immune phenotypes that can inform the development of preventive measures.

## INTRODUCTION

Enteropathogenic Escherichia coli (EPEC) and *Shigella* spp. are primary etiological agents of moderate to severe diarrhea (MSD) in children <5 years of age living in resource-poor countries (1). EPEC causes watery diarrhea, and although less prevalent than other diarrheagenic pathogens, it is associated with a higher risk of mortality in children aged 0 to 11 months with MSD (1, 2). *Shigella* spp. can cause dysentery, a more severe form of diarrhea accompanied by mucus and blood (1, 3). Acute diarrhea often causes a loss of fluid, resulting in severe dehydration that leads to death. The long-term consequences of the gut inflammation in chronic diarrhea include malnutrition and impaired growth and immunity (4).

The intestinal epithelial barrier prevents microbes residing in the intestinal lumen from accessing internal compartments. EPEC and *Shigella* trigger inflammatory processes in the human gut, accompanied by disruption of tight junctions and epithelial cell damage, all of which increase intestinal permeability (5, 6). EPEC binds to intestinal epithelial cells through intimin, a surface adhesion and virulence factor (7), and uses a type III secretion system (T3SS) to inject effector proteins that promote cell-to-cell spread. *Shigella* is transported across the intestinal epithelium through M (microfold) cells and is taken up by macrophages. It escapes intracellular death by inducing rapid apoptosis of macrophages and invades nearby epithelial cells, spreading across the intestinal epithelium, all via translocation of T3SS effectors (8, 9). Tissue-derived proinflammatory cytokines further recruit innate immune cells to the site of infection. While substantial progress has been made in understanding EPEC and *Shigella* pathogenesis, an accurate representation of mucosal immune responses in children following infection with these organisms is lacking, including pathogen engagement and interaction with the host’s mucosal immune cells and the ensuing innate and adaptive immune responses in the human gut.

The involvement of host immunity can impact the course and severity of disease. We hypothesized that *Shigella* and EPEC infection evoke unique immune responses in the human gut and that distinct mucosal immune profiles can be associated with severity of disease. To test this hypothesis, stool samples from EPEC- and *Shigella*-infected children enrolled in the Global Enteric Multicenter Study (GEMS) were investigated for the presence of proinflammatory cytokines, T_H_1 and T_H_2 cytokines, and the inflammatory mediators myeloperoxidase (MPO), calprotectin (CP), and lactoferrin (LF). Stools were also examined for the presence of IgG and IgA antibodies specific for EPEC intimin and *Shigella* lipopolysaccharides (LPS), invasion plasmid antigen B (IpaB), and virulence gene (VirG). The gastrointestinal immune phenotypes of *Shigella-* and EPEC-infected children were compared with those of controls with diarrhea due to unrelated pathogens and with those of controls without diarrhea. Pathogen-specific immune profiles were also analyzed based on severity of disease, i.e., EPEC infection with or without diarrhea and *Shigella* diarrhea with or without dysentery, to identify mucosal markers associated with illness that could be targets for future prophylactics and therapeutic treatments.

## RESULTS

### Cohort characteristics.

This study utilized stool samples from children that were part of the GEMS study (1), which aimed at elucidating attributable causes of MSD in children between 0 and 5 years of age. Individuals were classified as cases or controls based on criteria detailed in the GEMS study (1). Briefly, cases were children with MSD (i.e., 3 or more loose or watery stools within the last 24 h) and another health indicator from a prespecified list, while controls were children who had been free of diarrhea for at least 7 days before enrollment. Of 2,598 children enrolled from rural villages in the Upper River Region in The Gambia, 244 were included in the analysis presented here. The study population is summarized in Table 1. To evaluate the effect of *Shigella* and EPEC infection, the samples were divided into six groups based on the presence of *Shigella* or EPEC as determined by microbial culture techniques or PCR analysis (Table 1) (1). These included children with diarrhea that was culture-positive for *Shigella* with (group 1) or without (group 2) blood in stools (dysentery), children with EPEC-positive stool cultures with (group 3) or without (group 4) clinical diarrhea, children with diarrhea that was culture negative for both *Shigella* and EPEC (group 5), and children without diarrhea (group 6).

**TABLE 1:** Description of study population

Group	GEMS definition^*a*^	Blood in stool	No. in age range (mo)			M:F^*b*^	Total no. per group^*c*^
			0–11	12–23	24–59		
1. *Shigella*^*d*^ with dysentery	Case	+	2	21	7	1.1	30
2. *Shigella*^*d*^ without dysentery	Case	−	1	17	5	1.3	23
3. EPEC^*d*^ with diarrhea	Case/control	±	36	39	21	1.2	96^*f*^
4. EPEC^*d*^ without diarrhea	Control	±	14	27	8	1.9	49^*g*^
5. Diarrhea^*e*^	Case/control	−	9	7	4	1.2	20^*h*^
6. No diarrhea^*e*^	Control	−	9	7	10	1.0	26^*i*^

a GEMS cases had MSD, i.e., 3 or more loose or watery stools within the last 24 h, and another health indicator from a prespecified list. GEMS controls were free of diarrhea for at least 7 days before enrollment, but they could have developed diarrhea after enrollment.

b Ratio of the number of male (M) to female (F) subjects.

c For each biomarker where fewer than the total number were analyzed, random representative samples were examined due to the volume of remaining sample and/or assay availability.

d Identified by visual observation, standard microbial culture techniques, and PCR analysis.

e Negative for *Shigella* and EPEC (controls).

f *n* = 95 for cytokine analysis; *n* = 92 for MPO analysis.

g *n* = 42 for cytokine analysis; *n* = 43 for MPO analysis.

h *n* = 16 for cytokine analysis; *n* = 18 for MPO analysis.

i *n* = 25 for cytokine, MPO, and CP analyses.

### Proinflammatory cytokines associated with *Shigella* and EPEC diarrheal infection.

We first examined the presence of proinflammatory cytokines in stool extracts from *Shigella*- and EPEC-infected children as potential markers of mucosal immune activation evoked by these pathogens *in vivo*.

**FIG 1:**
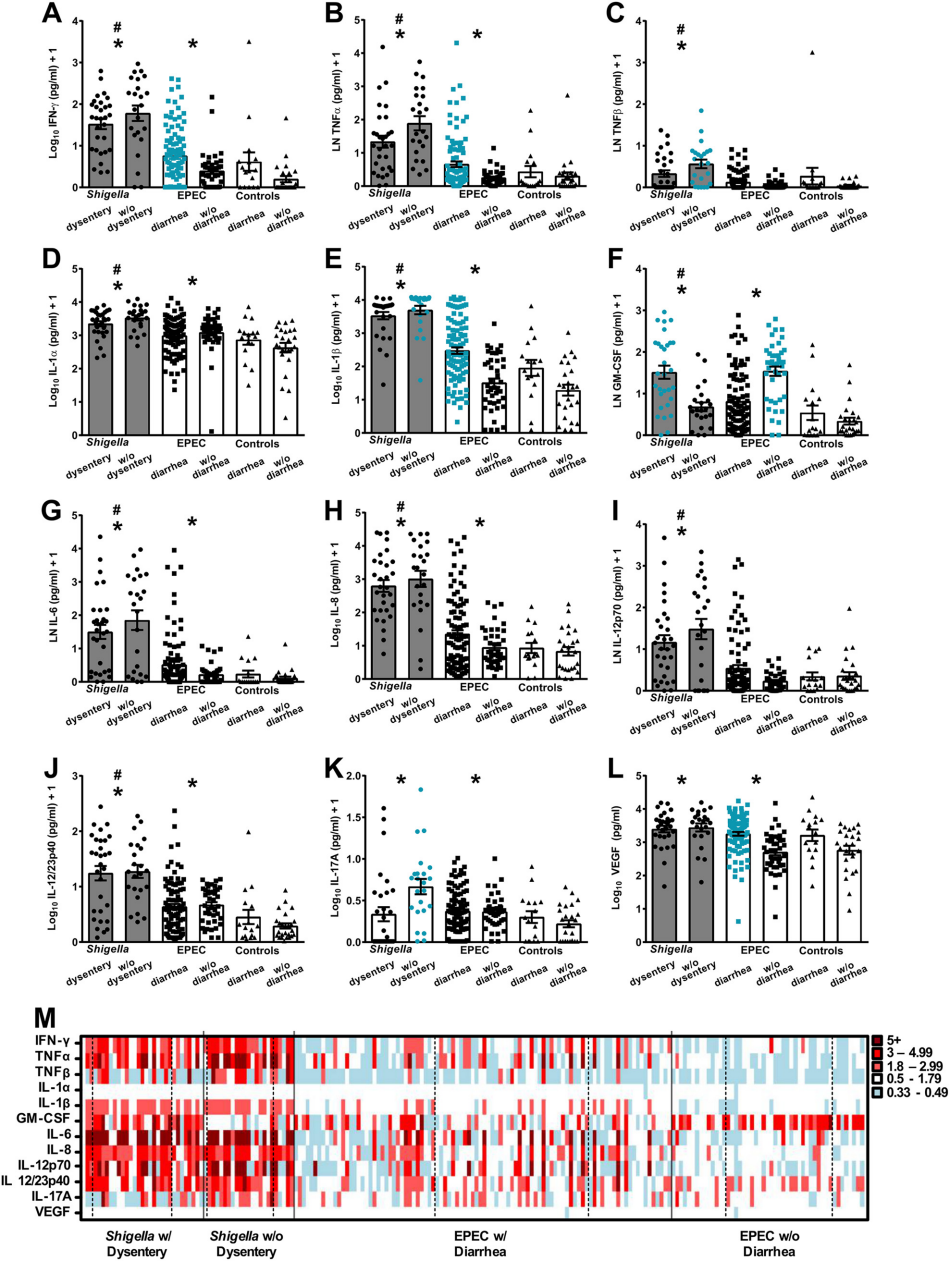
Proinflammatory cytokines quantified in stool supernatants. (A) IFN-γ, (B) TNF-α, (C) TNF-β, (D) IL-1α, (E) IL-1β, (F) GM-CSF, (G) IL-6, (H) IL-8, (I) IL-12p70, (J) IL-12/23p40, (K) IL-17A, and (L) VEGF were quantified in stool samples by Mesoscale Discovery multiplex immunoassays. Bars and error bars indicate means and standard errors of the means (SEM). Four levels of statistical comparisons were determined by rank regression pairwise analysis after correcting for age and sex differences and are shown in each plot: asterisks indicate significant differences comparing all samples from *Shigella-* and EPEC-infected children versus controls without diarrhea; pound signs indicate significant differences between all *Shigella-* or EPEC-infected children with diarrhea and the control group with diarrhea from other causes; gray shading indicates significant differences between *Shigella* infection (first two columns) compared to EPEC infection with diarrhea (third column); blue symbols indicate significantly higher responses when comparing *Shigella* with and without dysentery or EPEC with and without diarrhea. Differences in disease severity were determined by *t* tests of the parameter coefficients after adjustment for age and sex. A *P* value of ≤0.05 was considered significant. (M) Heat map indicates fold changes in proinflammatory cytokines (*y* axis) for each individual (*x* axis) relative to the average response of individuals with diarrhea from other causes. The left column indicates the immune response for the youngest child within that group, and age increases to the right. A solid vertical black line distinguishes the groups; a dotted vertical line indicates the different age ranges within each group (0 to 11 months [left], 12 to 23 months [middle], and 24 to 59 months [right]) as noted in Table 1.

Stools from children infected with *Shigella* (*n* = 53), all of whom had MSD (with or without dysentery), contained high levels of all of the proinflammatory cytokines measured: interferon gamma (IFN-γ), tumor necrosis factor alpha (TNF-α), TNF-β, interleukin 1α (IL-1α), IL-1β, granulocyte-macrophage colony-stimulating factor (GM-CSF), IL-6, IL-8, IL-12p70, IL-12/23p40, IL-17A, and vascular endothelial growth factor (VEGF). Cytokine levels in these children greatly surpassed the levels detected in children without diarrhea in the control group (*n* = 26) (Fig. 1; significant differences after adjustment for age and sex are indicated with asterisks). Likewise, several of these cytokines (IFN-γ, TNF-α, TNF-β, IL-1α, IL-1β, GM-CSF, IL-6, IL-8, IL-12p70, and IL-12/23p40) were elevated in *Shigella-*infected children above the level detected in children who had diarrhea due to other causes (*n* = 20) (Fig. 1; significant differences after adjustment for age and sex are indicated by pound signs). To ascertain differences in cytokine responses associated with disease severity, proinflammatory cytokine levels were compared between *Shigella*-infected children with and without dysentery. Children with dysenteric *Shigella* exhibited significantly lower levels of TNF-β, IL-1β, and IL-17A but higher levels of GM-CSF than those without dysentery after adjustment for age and sex (Fig. 1, blue circles).

The stools from EPEC-infected children (*n* = 145), with or without diarrhea, exhibited elevated levels of IFN-γ, TNF-α, IL-1α, IL-1β, GM-CSF, IL-6, IL-8, IL-12/23p40, IL-17A, and VEGF compared with children in the control group who did not have diarrhea after adjustment for age and sex (Fig. 1; differences are indicated with asterisks). No significant differences in proinflammatory cytokine responses were detected between EPEC-infected children (with or without diarrhea) after adjustment for age and sex and children with diarrhea due to other causes (see Table S1 in the supplemental material). The proinflammatory cytokine profiles were also compared based on disease severity; EPEC-infected children who exhibited diarrhea (*n* = 96) had higher levels of IFN-γ, TNF-α, IL-1β, and VEGF but lower GM-CSF levels than those without diarrhea after adjustment for age and sex (Fig. 1, blue squares).

10.1128/mbio.00538-22.6 TABLE S1 Pairwise comparison analysis *P* values. Download Table S1, DOCX file, 0.02 MB. Copyright © 2022 Buskirk et al. 2022 Buskirk et al. https://creativecommons.org/licenses/by/4.0/ This content is distributed under the terms of the Creative Commons Attribution 4.0 International license.

We next assessed the data set to identify proinflammatory cytokine patterns associated with pathogen-specific diarrhea. *Shigella*-positive MSD (*Shigella* cases with and without dysentery) was associated with increased amounts of all the cytokines measured except IL-17A (Fig. 1, gray filled columns) compared to EPEC with diarrhea. The complete comparison analysis (including all markers and groups) is shown in Table S1.

The individual proinflammatory cytokine response profile for all *Shigella-* and EPEC-infected children, shown as fold increase above the average response of children with diarrhea due to other causes, is summarized in a heat map array in Fig. 1M. To determine if cytokine levels were affected by the age of the children, heat maps are arranged by increasing age, with the response from the youngest child in the leftmost column within each group. Several findings stood out from this individual-data-point display: (i) a heightened proinflammatory response (red color) evoked by *Shigella* compared to EPEC infection, (ii) the elevated presence of TNF-β and IL-17A but absence of GM-CSF in *Shigella*-infected children who did not have dysentery compared to children who had *Shigella* with dysentery, (iii) increased production of proinflammatory cytokines when EPEC infection was accompanied by diarrhea, (iv) GM-CSF in the majority of EPEC-infected children who did not experience diarrhea, (v) distinctly elevated TNF-α and IL-12p70 levels in the 12- to 23-month and 24- to 59-month age groups of children with *Shigella* but no dysentery, and (vi) more pronounced inflammatory responses in children with diarrheagenic EPEC as they approached and into the second year of life.

### Inflammatory mediators induced during *Shigella* and EPEC infections.

**FIG 2:**
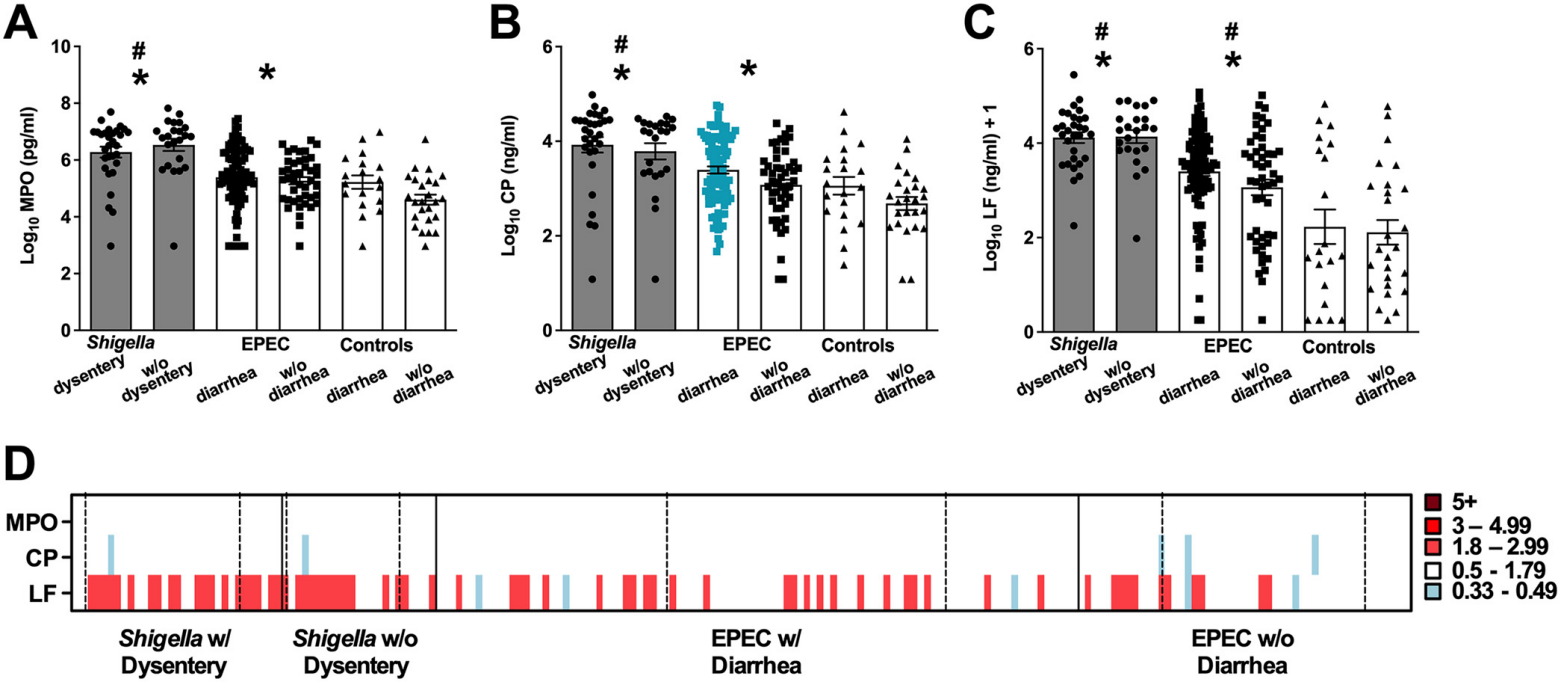
Inflammatory mediators quantified in stool supernatants. (A) Myeloperoxidase (MPO), (B) calprotectin (CP), and (C) lactoferrin (LF) were quantified in stool samples by Mesoscale Discovery singleplex immunoassays. Comparisons were performed as described in the legend to Fig. 1. Asterisks indicate significant differences comparing all samples from *Shigella-* and EPEC-infected children versus controls without diarrhea; pound signs indicate significant differences between all *Shigella-* or EPEC-infected children with diarrhea compared to the control group that had diarrhea from other causes; gray shading indicates significant differences between *Shigella* and EPEC infection with diarrhea. A *P* value of ≤0.05 was considered significant. (D) Heat map indicating fold changes in inflammatory mediators (*y* axis) for each individual (*x* axis) relative to the average of diarrhea from other causes. The left column indicates the immune response for the youngest child within that group, and age increases to the right. Solid vertical black lines distinguish the groups; dotted vertical lines indicate the different age ranges within each group (0 to 11 months [left], 12 to 23 months [middle], and 24 to 59 months [right]) as noted in Table 1.

We next measured the presence of inflammatory mediators MPO, CP, and LF (produced by intestinal epithelial cells, dendritic cells, and polymorphonuclear cells) as contributors to host innate immune defenses through their capacity to promote cell recruitment, phagocytosis, and microbial killing. Increased levels of MPO, CP, and LF were found in stools of both *Shigella-* and EPEC-infected children compared to children without diarrhea after adjustment for age and sex (Fig. 2, asterisks). *Shigella*-positive MSD evoked higher concentrations of MPO, CP, and LF than EPEC-positive diarrhea (Fig. 2, gray bars) and diarrhea due to other causes (differences are indicated by pound signs) after adjustment for age and sex. Diarrheagenic EPEC also resulted in significantly higher levels of LF than diarrhea from other causes. No differences in severity of disease for *Shigella* with and without dysentery were observed. However, children with diarrheagenic EPEC had significantly higher levels of CP than those with EPEC but no diarrhea after adjustment for age and sex (Fig. 2B, blue squares). Different from the proinflammatory cytokine responses, no overt differences in MPO, CP, or LF specific to pathogen, disease severity, or age were observed in the heat map array. Generally, however, lower LF levels were observed in the oldest children in the EPEC-without-diarrhea groups (Fig. 2D).

### T_H_1 and T_H_2 cytokine responses evoked during *Shigella* and EPEC infections.

**FIG 3:**
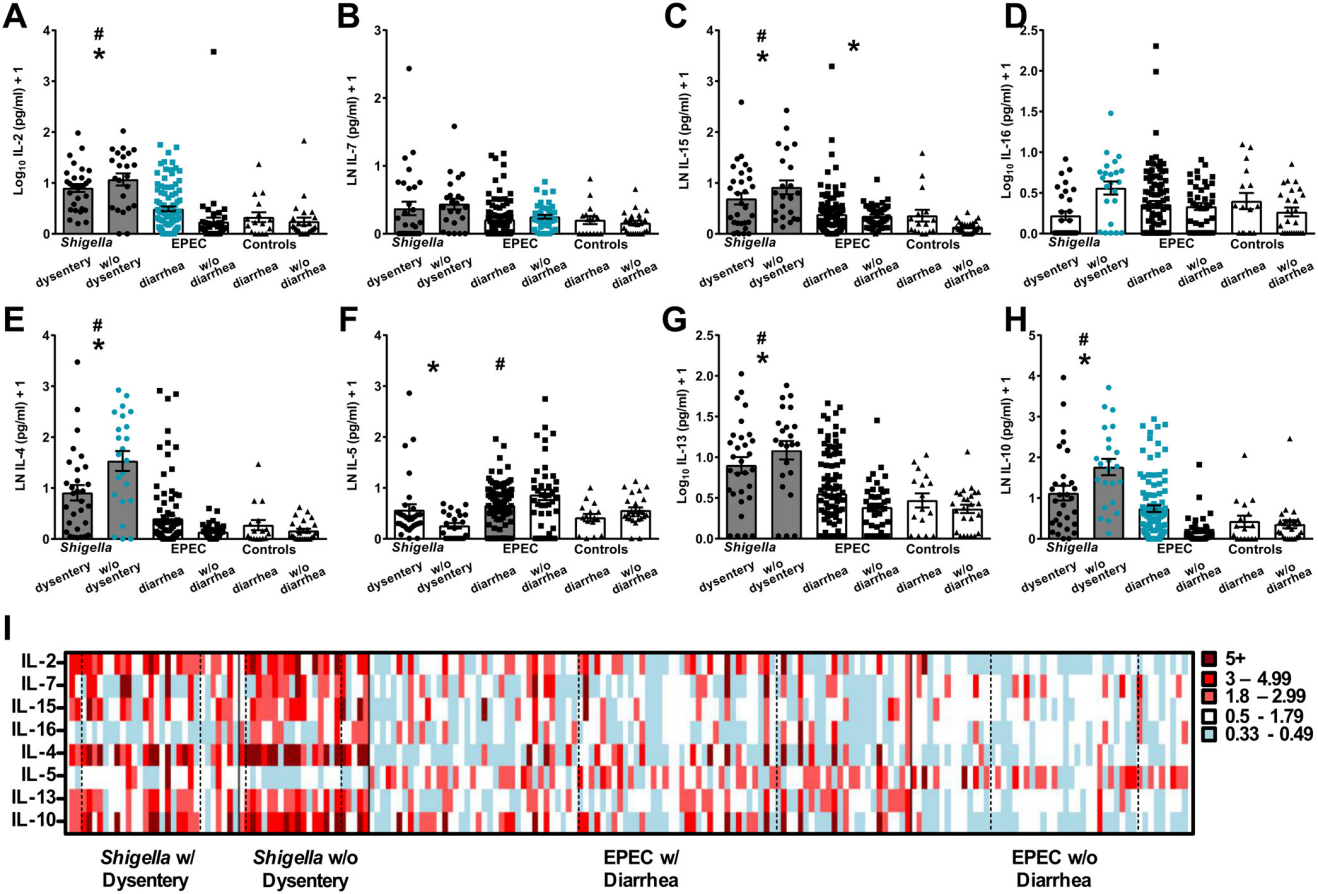
T_H_1 and T_H_2 cytokines in stool supernatants. (A) IL-2, (B) IL-7, (C) IL-15, (D) IL-16, (E) IL-4, (F) IL-5, (G) IL-13, and (H) IL-10 cytokines were quantified in individual samples by Mesoscale Discovery multiplex immunoassays. Statistical comparisons were performed as indicated for Fig. 1. Asterisks indicate significant differences comparing all samples from *Shigella-* and EPEC-infected children versus controls without diarrhea; pound signs indicate significant differences between all *Shigella-* or EPEC-infected children with diarrhea and the control group that had diarrhea due to other causes; gray shading indicates significant differences between *Shigella*-infected children (first two columns) and EPEC-infected children with diarrhea (third column). Blue symbols indicate significantly higher responses when comparing *Shigella* with and without dysentery or EPEC with and without diarrhea. A *P* value of ≤0.05 was considered significant. (I) Heat map indicating fold changes in T_H_1 and T_H_2 cytokines (*y* axis) for each individual (*x* axis) relative to the average for the group with diarrhea from other causes. The left column indicates the immune response for the youngest child within that group, and age increases to the right. A solid vertical black line distinguishes the groups; a dotted vertical line indicates the different age ranges within each group (0 to 11 months [left], 12 to 23 months [middle], and 24 to 59 months [right]) as noted in Table 1.

T_H_1 and T_H_2 cytokine responses were measured in the stools of *Shigella-* and EPEC-infected children as critical modulators of adaptive immunity. Children infected with *Shigella* had higher levels of IL-2, IL-15, IL-4, IL-5, IL-13, and IL-10 than controls without diarrhea after adjustment for age and sex (Fig. 3; differences are indicated by asterisks). The levels of IL-2, IL-15, IL-4, IL-13, and IL-10 were also increased in *Shigella-*infected children relative to those of children with diarrhea from other causes after adjustment for age and sex (Fig. 3; differences are indicated by pound signs). Children with dysenteric *Shigella* had lower levels of IL-16, IL-4, and IL-10 in their stools than children with *Shigella* without dysentery after adjustment for age and sex (Fig. 3, blue circles).

Among EPEC-infected children, only IL-15 levels were elevated above those of the no-diarrhea control group after adjustment for age and sex (Fig. 3, asterisks). Compared to children with diarrhea from other causes, diarrheagenic EPEC elicited increased levels of IL-5 after adjustment for age and sex (Fig. 3, pound signs). EPEC diarrhea was associated with higher production of IL-2 and IL-10 but lower IL-7 production compared with EPEC infection without diarrhea after adjustment for age and sex (Fig. 3; significantly higher values are indicated by blue squares).

Similar to what was observed for the proinflammatory cytokines, pathogen-specific T_H_1 and T_H_2 profiles associated with *Shigella* infection (all of whom had MSD, with or without dysentery) had higher levels of IL-2, IL-7, IL-15, IL-4, IL-13, and IL-10, but lower IL-5, compared to EPEC-infected children with diarrhea after adjustment for age and sex (Fig. 3, gray bars).

The individual cytokine profiles displayed in the heat map arrays showed (i) the capacity of *Shigella* to evoke a more vigorous mucosal T_H_1/T_H_2 cytokine response than EPEC, (ii) higher levels of IL-16 and IL-4 in children with less severe *Shigella* diarrhea, (iii) distinctly increased IL-5 levels in EPEC-infected children compared to those with diarrhea from other causes, and (iv) no evidence of age-specific differences or trends (Fig. 3I).

### Antibody responses during *Shigella* or EPEC infections.

**FIG 4:**
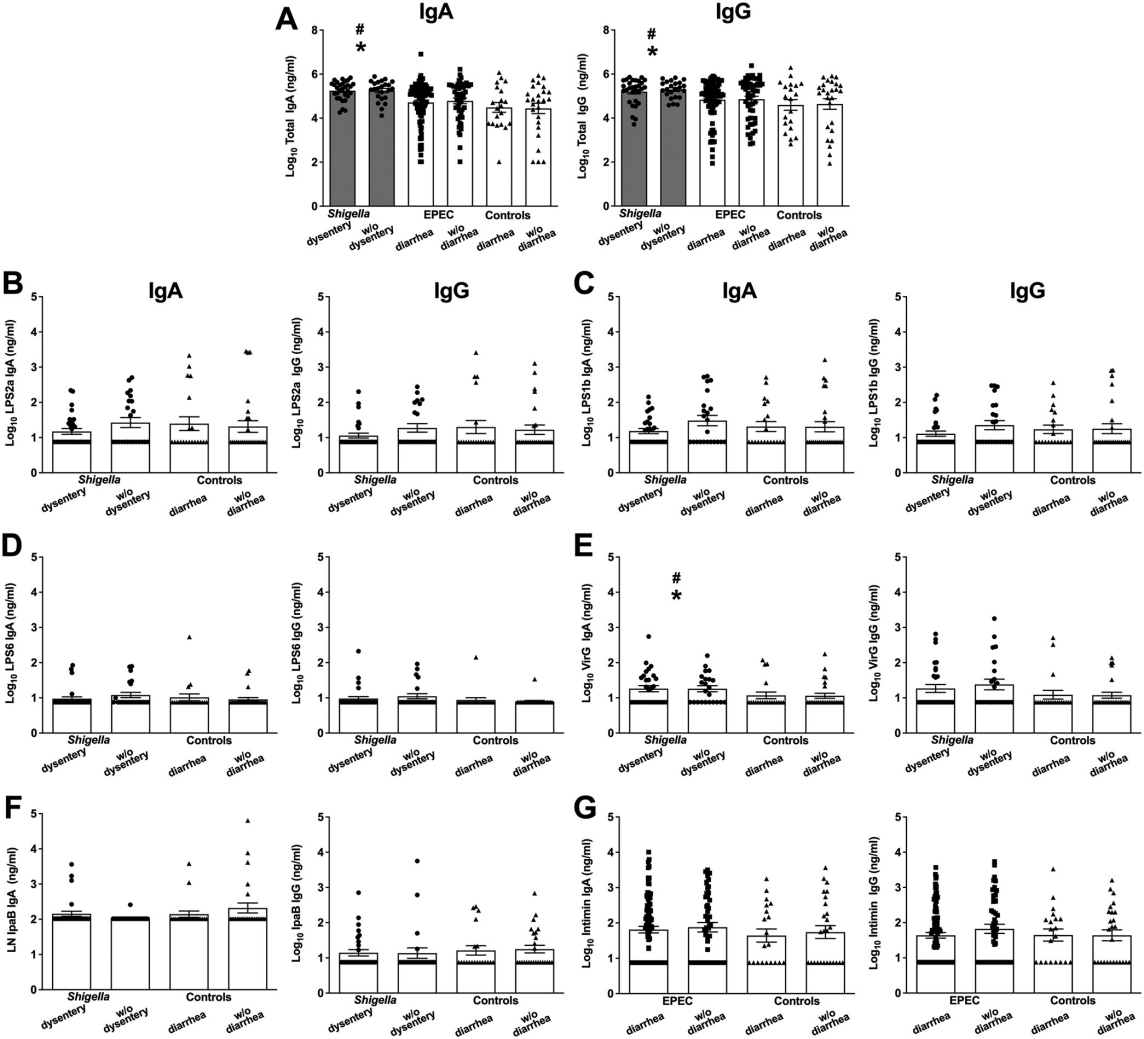
Antibody levels in stool supernatants. (A) Total and (B) LPS 2a-, (C) LPS 1b-, (D) LPS 6-, (E) VirG-, (F) IpaB-, and (G) intimin-specific IgA and IgG antibody levels in individual samples were measured by ELISA. Statistical comparisons were as indicated in Fig. 1. Asterisks indicate significant differences comparing all samples from *Shigella-* or EPEC-infected children versus controls without diarrhea; pound signs indicate significant differences between all *Shigella-* or EPEC-infected children with diarrhea and the control group with diarrhea due to other causes; gray shading indicates significant differences between *Shigella*-infected children (first two columns) and those with EPEC and diarrhea (third column). A *P* value of ≤0.05 was considered significant. No statistically significant differences were observed when comparing severity of disease for either pathogen.

IgG and IgA specific to key bacterial antigens, as well as total IgG and IgA, were also measured in the stool supernatants. Total IgA and total IgG were increased in *Shigella*-infected children compared to children without diarrhea (Fig. 4A, asterisks) and those with diarrhea from other causes (Fig. 4A, pound signs) after adjustment for age and sex. These antibodies were also higher in *Shigella*-infected children than in those with diarrheagenic EPEC (Fig. 4A, gray bars).

To investigate *Shigella*-specific immunity, we measured stool antibodies against conserved *Shigella* protein antigens, IpaB and VirG, and O antigens from the subspecies most prevalent in sub-Saharan Africa according to the GEMS study, i.e., Shigella flexneri 2a (LPS2a), 1b (LPS1b), and 6 (LPS6) (10). We found a trend, after adjustment for age and sex, of lower LPS2a- and LPS1b-specific IgA and IgG in children infected with *Shigella* and dysentery than in those with *Shigella* without dysentery and children with diarrhea from other causes, although the difference did not reach statistical significance (Fig. 4B and C). LPS6- and IpaB-specific IgA and IgG titers in children with *Shigella* infection were comparable to those in the controls (Fig. 4D and F). Significantly higher levels of VirG IgA were detected in *Shigella*-infected children after adjustment for age and sex than in children without diarrhea or children with diarrhea from other causes; VirG IgG followed the same trend, although it did not reach statistical significance (Fig. 4E). No significant differences were observed in antigen-specific antibodies between *Shigella*-infected children with and without dysentery (Fig. 4). It should be noted that with the exception of total IgA and total IgG, the antibody data set contained values below the limit of detection and thus should be interpreted cautiously.

To investigate EPEC-specific antibodies, we selected intimin as the target antigen; intimin is a classical EPEC virulence (adhesin) factor shared among both typical and atypical strains. After adjustment for age and sex, a trend of higher levels of intimin-specific antibodies in EPEC-infected children than those with nonspecific diarrhea and no-diarrhea controls was observed (Fig. 4G). Intimin IgG titers were also elevated in the EPEC-infected children without diarrhea compared to those with diarrheagenic EPEC, although the difference was not statistically significant (Fig. 4G).

### Pathogen-specific associated immune profiles.

**FIG 5:**
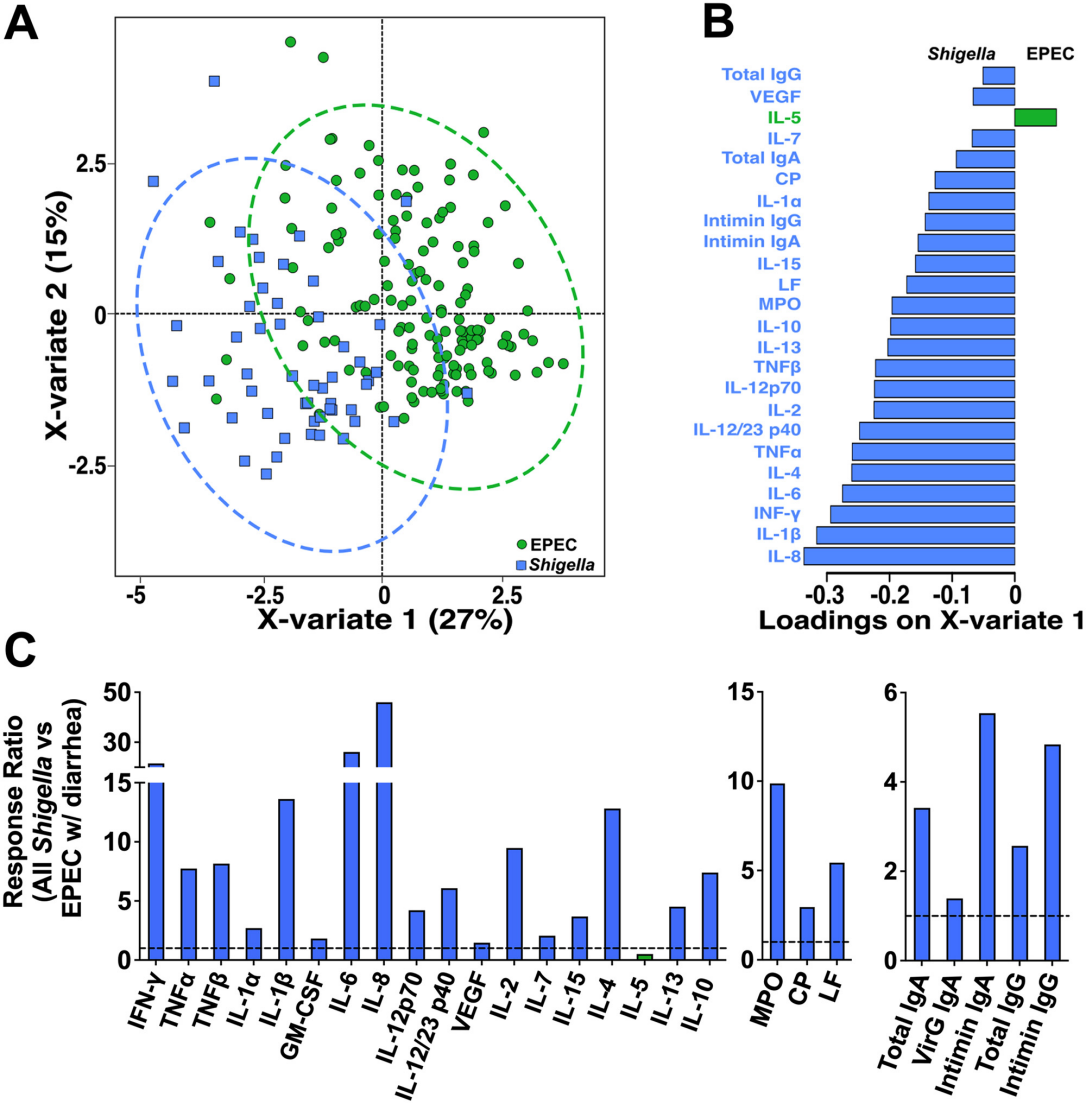
Pathogen-specific immune responses. (A) sPLS-DA depicting separation of immune responses between *Shigella*-infected and EPEC-infected individuals. Each symbol represents one individual, and the colors indicate the group. (B) Immune parameters that contributed significantly to *Shigella* infection (blue, pointing left) versus EPEC infection (green, pointing right), to X-variate 1, ranked based on loading score. (C) Response ratios of significantly different cytokines (left), inflammatory mediators (middle), and antibodies (right) were calculated by taking the geometric mean for all samples positive for *Shigella* and dividing by the geometric mean for samples from the EPEC-with-diarrhea group. The dotted line indicates a fold change of 1, or no difference between the groups. Bars below the dotted line show higher responses in the EPEC group, while bars above the line indicate higher responses in the *Shigella*-infected group.

To discern pathogen-specific immune profiles and identify biomarkers associated with pathogen-specific infection (*Shigella* or EPEC) or illness (dysentery or diarrhea), we conducted stepwise analyses. First, we performed a principal-component analysis (PCA) on all 37 immune parameters measured and compared immune profiles from all 6 groups (Table 1). We found a clear separation between immune response profiles of children with *Shigella* infection (with or without dysentery) and those of all other groups (Fig. S1). In contrast, there was no apparent segregation between EPEC-infected children and the controls. Second, we used a sparse partial least-squares discriminant analysis (sPLS-DA) to identify immune parameters that could discriminate *Shigella* versus EPEC infection (Fig. 5A and B). EPEC infection was associated with the presence of IL-5, while *Shigella* infection was associated with all other proinflammatory cytokines, inflammatory mediators, and total IgG and IgA (Fig. 5B). Interestingly, children infected with *Shigella* had EPEC-specific intimin fecal IgG and IgA. Lastly, we compared immune parameters that were statistically different between diarrheagenic *Shigella* and EPEC (gray bars in Fig. 1–4), this time using response ratios in all *Shigella*-infected children versus those infected with EPEC and exhibiting diarrhea. The results were consistent with the sPLS-DA analysis described above: *Shigella* infection was associated with substantial increases in almost all proinflammatory cytokine and inflammatory mediators (Fig. 5C, left and middle), with IL-5 being the only marker that was reduced. *Shigella* infection was also associated with increases in total IgA and IgG and in VirG IgA, and >5-fold-higher levels of intimin IgA and IgG compared with EPEC infection with diarrhea (Fig. 5C, right).

10.1128/mbio.00538-22.1 FIG S1 Group-specific immune responses ratios. (A) PCA performed on all 37 immune parameters measured for each sample. Each symbol represents one individual, and the colors indicate groups, as described in Table 1. Download FIG S1, TIF file, 0.3 MB. Copyright © 2022 Buskirk et al. 2022 Buskirk et al. https://creativecommons.org/licenses/by/4.0/ This content is distributed under the terms of the Creative Commons Attribution 4.0 International license.

Single-data-point heat map arrays show the markedly higher inflammatory responses associated with *Shigella* infection compared to diarrheagenic EPEC, which did not appear to be affected by age (Fig. S2).

### Disease severity-associated immune profiles.

**FIG 6:**
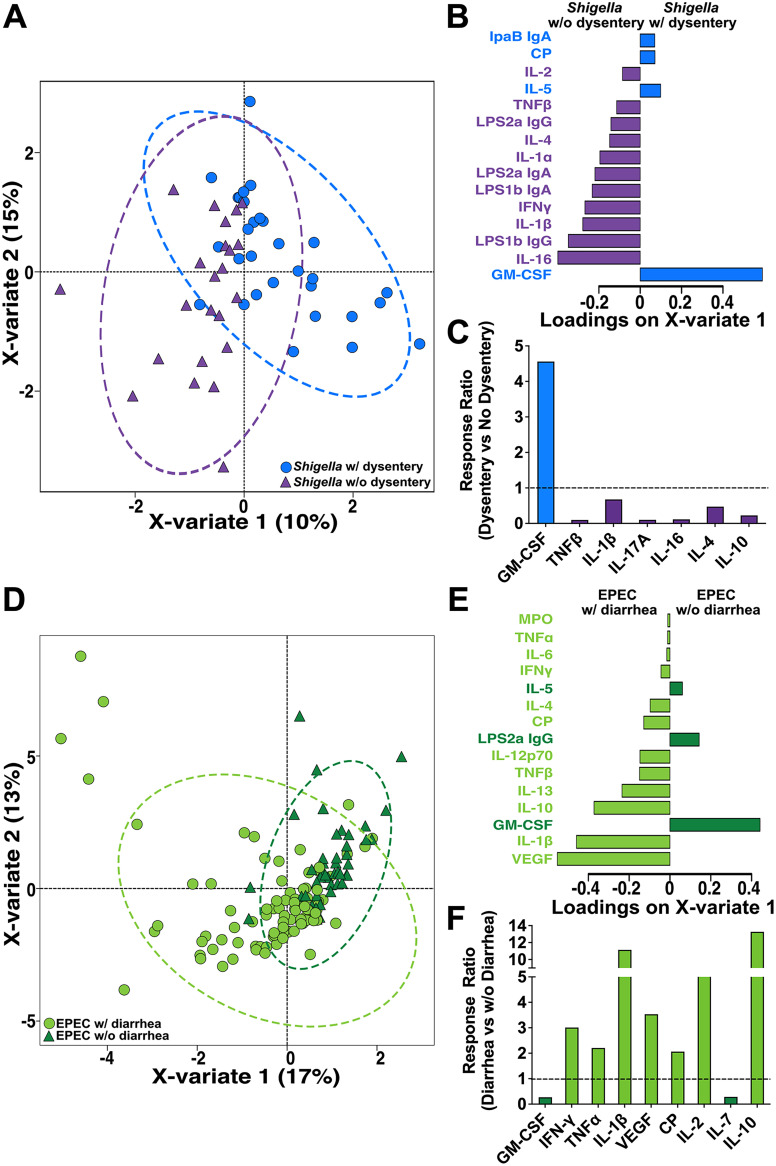
Immune profiles based on disease severity. (A) sPLS-DA to differentiate between *Shigella*-infected children with and without dysentery. (B) Immune parameters that contributed significantly to X-variate 1, ranked based on loading score, either to *Shigella* with dysentery (blue, pointing right), or to *Shigella* without dysentery (purple, pointing left). (C) Response ratios (mean cytokine levels in children with dysentery divided by mean levels in children without dysentery) for biomarkers that were significantly different between *Shigella*-infected children with and without dysentery. The dotted line represents a fold change of 1, indicating no change. (D) sPLS-DA to differentiate between EPEC-infected children with and without diarrhea. (E) Immune parameters that contributed significantly to X-variate 1, ranked based on loading score, either to EPEC with diarrhea (light green, pointing left) or to EPEC without diarrhea (dark green, pointing right). (F) Response ratios (mean cytokine levels in children with diarrhea divided by mean levels in children without diarrhea) for biomarkers that were significantly different between EPEC-infected children with and without diarrhea. The dotted line represents a fold change of 1, indicating no change.

To identify immune profiles associated with exacerbated disease for each of these pathogens, we performed sPLS-DA to identify markers that could discriminate between *Shigella* infection with or without dysentery and EPEC infection with or without diarrhea. Of the top 15 features that could segregate *Shigella* with dysentery and *Shigella* without dysentery (Fig. 6A and B), GM-CSF was the strongest immune marker for *Shigella* with dysentery, while the cytokines IL-16, IL-1β, and IFN-γ and antibodies against LPS1b (IgG and IgA) and LPS2a IgA had the strongest associations with absence of dysentery (Fig. 6B). Comparison of the response ratios of cytokines that were significantly different between children with and without dysentery confirmed that dysenteric *Shigella* was associated with >4-fold-higher levels of GM-CSF and reduced production of TNF-β, IL-1β, IL-16, and IL-4 (Fig. 6C).

A similar sPLS-DA analysis comparing features that could separate EPEC with diarrhea compared to EPEC without diarrhea (Fig. 6D and E) revealed that diarrheagenic EPEC was associated with proinflammatory cytokines, including VEGF, IL-1β, IL-2, and IL-10 (Fig. 6E). In contrast, GM-CSF, IL-5 and *Shigella*-specific LPS2a IgG were associated with EPEC without diarrhea (Fig. 6E). Comparison of the response ratios of cytokine/chemokines that were significantly different between children with and without diarrhea confirmed associations between EPEC with diarrhea and 2- to 13-fold increases in IFN-γ, TNF-α, IL-1β, VEGF, CP, and IL-10 and concomitant reductions in GM-CSF and IL-7 (Fig. 6F). Heat map arrays of immune markers associated with disease severity highlight the pathogen-specific differences in the stool immune markers based on disease severity (Fig. S3A and B). As indicated above, no age-specific trends were observed (Fig. S3A and B).

10.1128/mbio.00538-22.2 FIG S2 Pathogen-specific immune responses ratios. A heat map comparing the fold changes in all 37 parameters (y axis) for each *Shigella*-infected individual (x axis) with and without dysentery relative to EPEC with diarrhea. The left column indicates the immune response for the youngest child, and age increases to the right. Download FIG S2, TIF file, 1.3 MB. Copyright © 2022 Buskirk et al. 2022 Buskirk et al. https://creativecommons.org/licenses/by/4.0/ This content is distributed under the terms of the Creative Commons Attribution 4.0 International license.

10.1128/mbio.00538-22.3 FIG S3 Immune profile based on disease severity. A heat map indicating fold changes in all 37 parameters examined (*y* axis) for each individual (*x* axis) in *Shigella*-infected children with dysentery compared to the average of the *Shigella*-infected children without dysentery (A) or in EPEC-infected children with diarrhea group relative to the average of EPEC-infected children without diarrhea (B). The left column indicates the immune response for the youngest child, and age increases to the right. Download FIG S3, TIF file, 2.2 MB. Copyright © 2022 Buskirk et al. 2022 Buskirk et al. https://creativecommons.org/licenses/by/4.0/ This content is distributed under the terms of the Creative Commons Attribution 4.0 International license.

## DISCUSSION

Young children living in resource-poor countries are overburdened with diarrheal disease, with *Shigella* and enteropathogenic E. coli (EPEC) among the main attributable agents (1). Our limited understanding of host-microbe interactions, immune activation, and mechanisms underlying protective immunity have hindered progress in preventing these diseases. There is a lack of information on mucosal immune markers associated with *Shigella-* and EPEC-positive diarrhea and mucosal protective immune components that reduce severity or prevent infection in children. This study represents the first characterization of gut mucosal immune responses (i.e., stool cytokines, inflammatory mediators, and antibodies) in EPEC- and *Shigella-*infected children enrolled in the GEMS study and of pathogen-specific immunity based on disease severity (i.e., diarrhea and dysentery, respectively).

*Shigella* infection has been associated with extensive intestinal inflammation (11–14). Increased levels of IL-6 and TNF-α and higher numbers of white and red blood cells have been found in stools of 12- to 60-month-old Bangladeshi children with uncomplicated shigellosis (i.e., absence of hemolytic uremic syndrome and/or leukocytosis) in comparison to those with complicated disease, suggesting a protective role of these inflammatory mediators in preventing further bacterial spread and tissue damage (15). Increased levels of MPO and LF have also been reported in stools of 3- to 10-year-old Bangladeshi children experiencing acute disease (14). Neither of these studies related immunological outcomes with severity of diarrhea. In U.S. children infected with Shigella sonnei, increased IL-1β, IL-8, and CP mRNA transcripts were found in stool (16). These observations, however, were derived from a small group of children (only 7 cases of infected children and 3 controls) of unknown age range. Unique and important features of our study relative to those mentioned above include (i) the use of a large number of specimens from well-defined cases and controls, (ii) inclusion of children 0 to 59 months old representing the population most affected by diarrheal diseases, (iii) evaluation of stool samples obtained under stringent criteria (<72 h from onset of diarrhea), and (iv) comprehensive characterization of local inflammatory as well as adaptive immunity.

Strong proinflammatory and T cell responses distinguished *Shigella*-infected children from those infected with EPEC or with other pathogens. Their cytokine profile included elevated levels of IFN-γ, TNF-α, TNF-β, IL-1α, IL-1β, GM-CSF, IL-6, IL-8, IL-12p70, IL-12/23p40, and VEGF as well as T_H_1 (IL-2, IL-7, and IL-15) and T_H_2 (IL-4, IL-10, and IL-13) cytokines. The presence of proinflammatory cytokines, indicative of innate immune cell activation, likely reflects recent or acute infection, whereas T cell cytokines suggest a recall response from an earlier exposure to the organism. Increased levels of GM-CSF and reduced production of TNF-β, IL-1β, IL-17A, IL-16, IL-4, and IL-10 were distinct responses observed in *Shigella-*infected children with dysenteric diarrhea compared to *Shigella* without dysentery. Blood appears in stool as a result of intestinal cell damage and necrosis during *Shigella* infection, propelled by an abundance of infiltrating neutrophils (17). The heightened production of GM-CSF in the wake of dysentery may result from overwhelming granulocyte activation and may reflect a defense mechanism because GM-CSF can enhance neutrophil migration, delay apoptosis—possibly to prolong cell longevity—and activate T cells, all aimed at controlling infection (18). On the other hand, the reduction or absence of IL-17A in infected children who develop dysenteric disease reinforces the finding that this cytokine has a protective role, as established in mouse studies (19). A similar protective activity could be ascribed to the other three T-cell-derived cytokines reduced in dysenteric cases—IL-16 (chemoattractant of CD4 T cells) (20), IL-4 (induces T cell proliferation and differentiation into T_H_2 cells) (21), and IL-10 (regulates the inflammatory response to prevent extensive damage to the host and restricts T cell proliferation and differentiation) (22)—while their underlying mechanisms of action remain to be elucidated.

Unlike *Shigella* infection, EPEC infection does not induce excessive inflammation (7, 23). It is worth noting that some of the children infected with *Shigella* were coinfected with EPEC, and even then, the immune profile of the coinfection grouped with *Shigella* and not EPEC infection (Fig. S4). Therefore, the absence in our study of a distinct proinflammatory cytokine profile attributable to EPEC infection only was not surprising. Rather, the cytokine response profiles of EPEC-infected children appeared to be indistinguishable from those of children with diarrhea from other causes. Nevertheless, a distinct increase in MPO, CP, and LF was observed in EPEC-infected children, regardless of whether they had diarrhea, compared with control children without diarrhea. The higher levels of LF in EPEC-infected children compared to controls with diarrhea from other causes suggests that LF might reflect a host response aimed at controlling EPEC colonization, invasion, and neutralization of effector proteins *in vivo* (24).

10.1128/mbio.00538-22.4 FIG S4 PCA performed on all 37 immune parameters shows that the immune profile in children coinfected with both *Shigella* and EPEC clusters with the profile in *Shigella*-infected, and not EPEC-infected, children. Download FIG S4, TIF file, 0.3 MB. Copyright © 2022 Buskirk et al. 2022 Buskirk et al. https://creativecommons.org/licenses/by/4.0/ This content is distributed under the terms of the Creative Commons Attribution 4.0 International license.

When cytokine responses were compared considering disease severity, EPEC-positive diarrhea was associated with higher levels of IFN-γ, TNF-α, and IL-1β, known to promote innate immune cell recruitment, and T-cell-derived IL-2 and IL-10, which stimulate and control CD4^+^ T helper responses, respectively. IL-10 has also been shown to inhibit LPS-induced production of proinflammatory cytokines, including IFN-γ, TNF-α, IL-1β, IL-6, IL-12, and IL-15 (25–28). VEGF was elevated in children with EPEC-positive diarrhea; this molecule, produced by epithelial cells (29) and innate granulocytes, has pleiotropic and mostly host-protective functions (30). The elevated levels of CP in the stools of children with diarrheagenic EPEC suggests neutrophil recruitment to the intestinal lumen to aid in clearance of the pathogen; CP is a metal chelator that removes environmental calcium, zinc, and manganese required for microbial growth (31–33). Strikingly, GM-CSF levels were lower in children with diarrheagenic EPEC infection than in those with EPEC but without diarrhea. Considering the limited involvement of neutrophils and inflammatory cells during EPEC infection compared to *Shigella*, it can be argued that GM-CSF in stool might reflect innate immune activation (less abundant in children with EPEC-positive diarrhea) as opposed to a protective mechanism. The lack of IL-7, produced by intestinal epithelial cells to promote T and B cell development, in children with EPEC-associated diarrhea further supports a limited immune activation during acute EPEC infection or the previously acquired immunity (34, 35). The absence of these cytokines may also reflect organism-specific invasion tactics or host adaptations.

A study of EPEC-infected Brazilian infants 5 weeks to 15 months old reported elevated levels of IFN-γ, TNF-α, IL-6, IL-4, and IL-10 in stools obtained 1 week after diarrheal onset (36). The differences in children’s age (up to 15 months versus 5 years in our study), timing of sample collection, which is critical for postinfection analysis (1 week versus up to 72 h onset in our study), and outcomes examined (duration of diarrhea versus presence or absence/onset in our case) preclude direct comparison of its data with ours. Notwithstanding, a similar cytokine profile associated with symptomatic EPEC infection emerged from both studies that implicates both T_H_1 and T_H_2 responses. The elevated level of IL-10 in these children is noteworthy and highlights (as in the case of *Shigell*a) the host’s effort to control inflammation and prevent intestinal epithelial damage during early stages of infection.

Cooperating with innate immune cells, mucosal antibodies represent a critical adaptive immune defense mechanism in the gut. A few studies reported very low levels or absence of antibodies against *Shigella* in stool of children living in regions of endemicity (37–39). *Shigella-*specific IgG and IgA were detected by Western blot analysis in the stools and duodenal contents of a small number of 1- to 62-month-old Peruvian children (37); no antibody concentrations were reported, nor were LPS-specific antibodies (known to be an important marker associated with reduced risk of disease and clinical protection) measured (40, 41). Reduced total IgA levels have been reported in stools of 1- to 5-year-old Bangladeshi children with complicated shigellosis compared to those with uncomplicated illness, with no differences detected in LPS-specific IgA titers (38). A novel contribution of our study was the analysis of total as well as antigen-specific stool IgG and IgA levels in *Shigella*-infected children. The specificity repertoire we examined included virulence factors important for *Shigella* pathogenesis (IpaB and VirG) as well as the serotype-specific antigens LPS2a, LPS1b, and LPS6 from subspecies prevalent in sub-Saharan Africa (10), all presumed to have a protective role based on previous reports (41). Children with diarrhea from causes other than *Shigella* had generally higher levels of antigen-specific antibodies than those in the *Shigella*-with-dysentery group, which is consistent with these antibodies having a protective role. Timing of sampling (after infection) can impact antibody determinations; LPS-specific IgA peaks 8 to 10 days after onset of symptoms (39), which would have been missed in our specimens collected 1 to 3 days after infection. Children from groups not affected by *Shigella* may have acquired immunity from prior exposure.

There is only one report in the literature on mucosal antibodies in duodenal lavage specimens from EPEC-infected children aged 4 weeks to 15 months living in the United Kingdom (42); the results are limited to presence or absence of agglutination of the stool-recovered strain from each child at the time of clinical presentation and 4 to 14 days after symptom onset. To our knowledge, ours is the first determination of EPEC-specific IgG and IgA content in stools of EPEC-infected and noninfected controls. Among many bacterial antigens, intimin was of particular interest because of its role in bacterial attachment and relevance as a vaccine candidate; an intimin-based vaccine has been successfully used in livestock to prevent EPEC infections (43, 44). Interestingly, intimin antibody levels were higher in children infected with *Shigella* than in both controls and EPEC-infected children; the relevance of this observation is not clear from our study and requires further investigation.

It has been hypothesized that young children are more susceptible to disease due to the inability of the uneducated immune system to mount an efficient proinflammatory response. To examine any age-specific trends, we presented individual immune responses in the form of heat map arrays ordered by increasing age. In general, no distinct differences in the quantified immune markers were noted in comparisons of children grouped as shown in Table 1: 0 to 11 months, 12 to 23 months, and 24 to 59 months. This suggests that even very young children have the immune capacity to respond to an enteric infection in a pathogen-specific manner. Of interest, we had observed more robust cytokine responses in children with diarrheagenic EPEC after the first year (Fig. 1). The immune profile of 0- to 11-month-old EPEC-infected children without diarrhea (compared to that of 12- to 59-month-old children) included IgG and IgA against intimin (and multiple other antibodies), IL-2, IL-5, TNF-α, TNF-β, IL-15, and CP (Fig. S5B). In contrast, these markers were absent or greatly reduced in 0- to 11-month-old children with diarrheagenic EPEC (Fig. S5A). The differences in biomarkers may reflect a host response aimed at controlling EPEC infection that is lacking in children with more severe disease. A previous analysis of the overall GEMS cohort found an increased risk of death in case infants with typical EPEC aged 0 to 11 months (1). A reduced antimicrobial immune profile in infants infected with diarrheagenic EPEC could explain their vulnerability and heightened risk of negative outcomes. The sample size in the different age ranges in our cohort varied; therefore, these results await confirmation.

10.1128/mbio.00538-22.5 FIG S5 Immune profiles based on age group. PLS-DA discriminates immune profiles in EPEC-infected children 0 to 11 months old (blue) versus those 12 to 59 months old (orange) with (A) or without (B) diarrhea. Bar graphs show immune parameters that contributed significantly to X-variate 1, ranked based on loading score, either to 0- to 11-month-old (blue) or to 12- to 59-month-old (orange) children. Download FIG S5, TIF file, 0.7 MB. Copyright © 2022 Buskirk et al. 2022 Buskirk et al. https://creativecommons.org/licenses/by/4.0/ This content is distributed under the terms of the Creative Commons Attribution 4.0 International license.

Despite being phylogenetically related, *Shigella* and EPEC are clearly different in their host-pathogen interactions, pathogenicity, engagement of the immune system, and triggering of immunological effectors. Infection with *Shigella* resulted in increased levels of nearly all the immune markers we measured compared with diarrheagenic EPEC and even diarrhea from other causes, including viruses (rotavirus, norovirus, sapovirus, astrovirus, and adenovirus), suggesting heavy reliance on innate immune cell recruitment during early stages of infection. In contrast, EPEC pathogenesis bypasses innate immunity. It attracted our attention that IL-5 was elevated in EPEC-infected children compared to all other groups. Because of IL-5’s association with parasitic infections (45), we examined the presence of parasites in each group and found parasitic coinfections in 30.7% of the children examined, many of whom did not have diarrhea (data not shown). Interestingly, a *post hoc*-analysis study of GEMS showed that Giardia lamblia infections were more closely associated with a decreased, rather than an increased, risk of diarrheal diseases in toddlers (46); however, no significant differences in the frequency of parasitic coinfections were observed between *Shigella-* and EPEC-infected children. This would imply that IL-5 might be a strategy used by EPEC to dampen inflammation during early infection.

The biomarker-disease association analysis conducted here was exploratory. It would be important to confirm and expand the results obtained in future studies. In conclusion, we described, for the first time, distinct immune profiles associated with severity of *Shigella* and EPEC diarrheal disease in children in regions of endemicity. These results provide the foundation for further analysis of host-microbe interactions during infection and mechanisms of protective immunity.

## MATERIALS AND METHODS

### Study samples.

The study included stool samples from 244 children (0 to 59 months of age) enrolled in the GEMS study at the Upper River Region in The Gambia. The samples were divided into six groups representing different disease severities and were based on GEMS information at and after stool collection (1); a complete description of the study population is provided in Table 1. All the samples from GEMS cases indicated that the child had MSD, i.e., 3 or more loose or watery stools within the last 24 h, and another health indicator from a prespecified list. GEMS controls were free of diarrhea for at least 7 days before enrollment, but they could have developed diarrhea after enrollment. The enrollment requirements for diarrhea cases and their age-matched controls were described in detail elsewhere (1). Fresh stool samples were obtained at enrollment (within 3 days of symptom onset) and stored at −80°C. The presence of etiological agents, particularly *Shigella* and EPEC, was tested by conventional microbial culture, multiplex PCR, and/or commercial immunoassays (47). The stool samples used in this study were obtained from the MRC Unit repository in The Gambia, based on the amount necessary for the assays (at least 1 g available). Children with MSD and stools positive for *Shigella* (all GEMS cases) were classified based on the presence or absence of blood in the stools (as reported by the child’s caretaker and/or laboratory assessment). The remaining children with EPEC-positive stool cultures were classified according to the presence or absence of clinical diarrhea; this group contained GEMS MSD cases and controls who went on to develop diarrhea within 5 days after enrollment. Of the remaining samples, those with no diarrhea for 7 days prior to and at least 5 days after enrollment were classified as no-diarrhea controls. Children with diarrhea that was culture negative for both *Shigella* and EPEC, including both MSD cases and GEMS controls who developed diarrhea within 5 days after enrollment, made up the “diarrhea from other causes” control group. Stool samples were shipped frozen to the University of Maryland, Baltimore.

### Stool extracts.

A 300-mg aliquot of each stool sample was placed in preweighed tubes containing ~1.5 g of 2.3-mm zirconium beads (Biospec, Bartlesville, OK) and 1 mL of extraction buffer (phosphate-buffered saline [PBS; pH 7.4] containing 0.01% soybean trypsin inhibitor, 0.1% EDTA, 0.5% phenylmethanesulfonyl fluoride solution, and 0.05% Tween 20, all from Sigma, St. Louis, MO). Stool samples were subjected to three 1-min beating cycles in a mini-Beadbeater-8 tissue homogenizer (Biospec, Bartlesville, OK) with 2-min incubations on ice between cycles and centrifuged at 14,000 rpm for 30 min at 4°C. The supernatant was collected, 10 μL of 1% bovine serum albumin containing 0.1% sodium azide (vol/vol) (Sigma) was added, and the mixture was stored at −80°C until use.

### Total and antigen-specific IgG and IgA enzyme-linked immunosorbent assays (ELISAs).

For total IgA or IgG measurements, Immulon II plates (Fisher Scientific, Pittsburgh, PA) were coated with purified anti-IgA (α-chain specific) or anti-IgG (γ-chain specific; Jackson Immunoresearch, West Grove, PA) at 1 μg/mL in PBS for 3 h at 37°C. To measure antigen-specific antibodies, Immulon II plates were coated with *Shigella* LPS2a, -1b, or -6 at 5 μg/mL, IpaB at 0.1 μg/mL in PBS, or VirG or E. coli intimin at 2 μg/mL in carbonate buffer, pH 9.6. After coating, plates were washed with PBS containing 0.05% Tween 20 (PBST) and blocked overnight at 4°C with PBS containing 10% nonfat dry milk (Nestle, Glendale, CA). Stool supernatant samples were added to the plates and serially diluted in PBST containing 10% nonfat dry milk (PBSTM) starting at 1:1,000 for total IgA and IgG or at 1:50 for antigen-specific IgG and IgA measurements. After a washing in PBST, bound antibodies were detected by incubating plates for 1 h at 37°C with biotinylated goat anti-human Fc-specific IgA or IgG (Jackson Immunoresearch) diluted 1:10,000 in PBSTM. Plates were washed and incubated at 37°C for 30 min with avidin peroxidase (Sigma) diluted 1:200 in PBSTM. Tetramethylbenzidine (TMB; KPL, Gaithersburg, MD) was added as the substrate for 15 min in the dark with shaking, and the reaction was stopped by adding 100 μL of 1 M phosphoric acid (Sigma). The amount of total and antigen-specific IgA and IgG in each sample was determined by extrapolation in standard curves of purified human IgA or IgG (Calbiochem, Madison, WI).

### Cytokine analysis.

Cytokines were quantified using proinflammatory panel I and cytokine panel I V-Plex multiplex immunoassays (Meso Scale Discovery, Bethesda, MD) according to the manufacturer’s protocol. Samples were tested at a 1:2 or 1:5 dilution in 10% blocker A solution, in duplicate. Plates were read using the QuickPlex SQ 120 instrument (Meso Scale Discovery). The concentration of each analyte was determined using the Meso Scale Workbench software v4.0.12.

### Inflammatory mediators.

MPO was quantified using a multiplex commercial assay according to the manufacturer’s protocols (Meso Scale Discovery). CP was quantified using a prototype Meso Scale Discovery assay (Meso Scale Discovery) including a human CP as standard (Hycult, Plymouth Meeting, PA). LF was quantified using a singleplex assay developed in-house. Briefly, standard 96-well, single-spot electrochemiluminescent plates (Meso Scale Discovery) were coated overnight at 4°C with antilactoferrin monoclonal antibody (clone 2B8; Abcam, Cambridge, MA) at 4 μg/mL in sterile PBS, pH 7.4. Plates were blocked with blocker A (Meso Scale Discovery) solution for 2 h at room temperature with shaking at 250 rpm and then washed with PBST. Samples (diluted 1:1,000 in 10% blocker A) were added to the plate and incubated for 1 h at room temperature. SULFO-tag (Meso Scale Discovery)-labeled mouse monoclonal anti-lactoferrin antibody (KT33; Novus Biologicals, Littleton, CO), at 1 μg/mL in 10% blocker A, was used as secondary antibody. After a washing, surfactant-free Tris-based read buffer containing tripropylamine (Meso Scale Discovery) was added to each plate immediately before reading on a QuickPlex SQ 120. Concentrations were determined using a standard curve of purified human LF (Sigma).

### Statistical analysis.

Analyte concentrations that were below detection levels were assigned a value that was one-half the lower limit of quantification. Data points were log transformed for graphical representation. A log_10_ transformation was used for markers that spanned a wide concentration range with multiple observations greater than 100 pg/mL. Natural log (ln) was used for biomarkers whose upper limit was typically below 100 pg/mL. IL-17A and IpaB IgG levels were ln transformed for better representation of the data distribution. If the variable contained values below 1, a 1 was added to the all the data prior to log transformation so that all log values would be in the positive range.

Immune markers from the different study groups were compared using pairwise comparisons among the following groups. (i) Immune markers in stools from children infected with *Shigella*, children infected with EPEC, and control children without diarrhea were compared. (ii) Immune markers in children with *Shigella* dysentery, children with EPEC diarrhea, and children with diarrhea from other causes were compared. (iii) Markers in stools of children with *Shigella* and dysentery were compared to those of children with *Shigella* without dysentery. (iv) Immune markers in children with EPEC and diarrhea were compared to those in children infected with EPEC without diarrhea. Rank regression of immune biomarkers with adjustment for age (in months) and sex was used to compare groups. Because *Shigella* is known to infect older children, we adjusted for age and sex, regardless of its significance in the rank regression. *P* values from a *t* test of the estimated parameter coefficients from the rank regression corresponding to more severe disease were used to determine whether there was a difference in biomarker expression after adjustment for sex and age. No adjustments for multiple comparisons were made due to the exploratory nature of this analysis, as has been previously described (48–50). Data were analyzed using SAS 9.4 (Cary, NC) and GraphPad Prism (San Diego, CA), and results with *P* values of <0.05 were considered statistically significant.

Immune marker heat maps were constructed using the ratio of response for each subject to the average response of the comparative group (using log-transformed values). For graphical representation of immune markers, response ratios were expressed as geometric means.

Ordination analysis to identify immune parameters associated with samples from children in the different groups was performed using principal-component analysis (PCA) on log-transformed values for each parameter. Plots were generated using GraphPad Prism 9. Sparse partial least-squares discriminant analysis (sPLS-DA) to reduce multivariate dimensionality (51–53) was performed using the mixOmics Bioconductor package (version 3.14) (54).
