# Supplementary material for: Mucosal Immune Profiles Associated with Diarrheal Disease Severity in Shigella- and Enteropathogenic Escherichia coli-Infected Children Enrolled in the Global Enteric Multicenter Study
Source: mBio. 2022 Aug 4;13(4):e00538-22. doi: 10.1128/mbio.00538-22 (PMC9426439; doi:10.1128/mbio.00538-22)
Supplement: TABLE S1 [file mbio.00538-22-s0006.docx]

| Supplementary Table S1. Pairwise comparison analysis p values | | | | | | | |
| --- | --- | --- | --- | --- | --- | --- | --- |
|  | ***Shigella*-infected children** | | | | **EPEC-infected children** | | |
| Immune Marker | **vs. EPEC-infected children with diarrhea** | **vs. children without diarrhea** | **vs. children with diarrhea from other causes** | **with dysentery vs.**  **children**  **without dysentery** | **vs. children without diarrhea** | **with diarrhea vs. children with diarrhea from other causes** | **with diarrhea vs.**  **children without diarrhea** |
| IFN-γ | <.0001 | <.0001 | <.0001 | 0.1681 | 0.0002 | 0.4714 | 0.0019 |
| TNFα | <.0001 | <.0001 | <.0001 | 0.0570 | 0.0945 | 0.1653 | 0.0085 |
| TNFβ | <.0001 | 0.0004 | 0.0572 | 0.0415 | 0.5689 | 0.9908 | 0.5503 |
| IL-1α | <.0001 | <.0001 | 0.0002 | 0.1412 | 0.0105 | 0.5935 | 0.3373 |
| IL-1β | <.0001 | <.0001 | <.0001 | 0.0192 | <.0001 | 0.1261 | <.0001 |
| GM-CSF | 0.0147 | <.0001 | 0.0032 | 0.0004 | <.0001 | 0.1450 | <.0001 |
| IL-6 | <.0001 | <.0001 | <.0001 | 0.3473 | 0.0190 | 0.1545 | 0.0823 |
| IL-8 | <.0001 | <.0001 | <.0001 | 0.3170 | 0.0876 | 0.4168 | 0.2270 |
| IL-12p70 | <.0001 | 0.0004 | 0.0024 | 0.4117 | 0.9232 | 0.7359 | 0.1668 |
| IL-12/23p40 | <.0001 | <.0001 | <.0001 | 0.9636 | 0.0001 | 0.1748 | 0.4864 |
| IL-17A | 0.7144 | 0.0288 | 0.4943 | 0.0005 | 0.0249 | 0.6111 | 0.8937 |
| VEGF | 0.0315 | <.0001 | 0.3757 | 0.4750 | 0.0404 | 0.9980 | <.0001 |
| MPO | <.0001 | <.0001 | <.0001 | 0.3568 | 0.0012 | 0.7105 | 0.2928 |
| CP | <.0001 | <.0001 | <.0001 | 0.3169 | 0.0014 | 0.3012 | 0.0258 |
| LF | <.0001 | <.0001 | <.0001 | 0.7143 | 0.0001 | 0.0483 | 0.1330 |
| IL-2 | <.0001 | <.0001 | <.0001 | 0.1745 | 0.2188 | 0.3564 | 0.0001 |
| IL-7 | 0.0249 | 0.1531 | 0.3096 | 0.1551 | 0.8003 | 0.9996 | 0.0236 |
| IL-15 | <.0001 | <.0001 | 0.0087 | 0.1963 | 0.0013 | 0.8915 | 0.4193 |
| IL-16 | 0.8595 | 0.6110 | 0.9408 | 0.0003 | 0.6444 | 0.8873 | 0.8343 |
| IL-4 | <.0001 | <.0001 | 0.0002 | 0.0193 | 0.4889 | 0.8930 | 0.1544 |
| IL-5 | <.0001 | 0.1032 | 0.7407 | 0.0557 | 0.3095 | 0.1124 | 0.1526 |
| IL-13 | <.0001 | <.0001 | 0.0044 | 0.1812 | 0.4414 | 0.8262 | 0.0803 |
| IL-10 | <.0001 | <.0001 | <.0001 | 0.0109 | 0.6881 | 0.2062 | <.0001 |
| Total IgA | <.0001 | 0.0003 | 0.0001 | 0.8847 | 0.7575 | 0.5750 | 0.3845 |
| LPS2a IgA | 0.7229 | 0.6025 | 0.8329 | 0.3100 | 0.6060 | 0.6798 | 0.1988 |
| LPS1b IgA | 0.4229 | 0.3766 | 0.5478 | 0.2988 | 0.9115 | 0.8224 | 0.0500 |
| LPS6 IgA | 0.2633 | 0.4148 | 0.6386 | 0.1971 | 0.9750 | 0.9929 | 0.3414 |
| VirG IgA | 0.0078 | 0.0430 | 0.0511 | 0.6585 | 0.8807 | 0.9001 | 0.7013 |
| IpaB IgA | 0.3387 | 0.3680 | 0.9997 | 0.2437 | 0.5668 | 0.8207 | 0.1611 |
| Intimin IgA | <.0001 | 0.0003 | 0.0002 | 0.4319 | 0.9462 | 0.7986 | 0.6700 |
| Total IgG | 0.0005 | 0.0809 | 0.0071 | 0.9672 | 0.9702 | 0.8154 | 0.4543 |
| LPS2a IgG | 0.9999 | 0.9816 | 0.9147 | 0.2343 | 0.9262 | 0.9107 | 0.4049 |
| LPS1b IgG | 0.8050 | 0.8093 | 0.9394 | 0.2032 | 0.7664 | 0.8647 | 0.5500 |
| LPS6 IgG | 0.3831 | 0.1502 | 0.3171 | 0.4655 | 0.6022 | 0.6334 | 0.2182 |
| VirG IgG | 0.5895 | 0.2567 | 0.2351 | 0.3291 | 0.0914 | 0.0904 | 0.9388 |
| IpaB IgG | 0.8832 | 0.3944 | 0.8331 | 0.4896 | 0.6392 | 0.7419 | 0.0119 |
| Intimin IgG | <.0001 | 0.0009 | 0.0010 | 0.9488 | 0.9843 | 0.9996 | 0.2847 |
