# Supplementary figures and images for: Mucosal Immune Profiles Associated with Diarrheal Disease Severity in Shigella- and Enteropathogenic Escherichia coli-Infected Children Enrolled in the Global Enteric Multicenter Study
Source: mBio. 2022 Aug 4;13(4):e00538-22. doi: 10.1128/mbio.00538-22 (PMC9426439; doi:10.1128/mbio.00538-22)

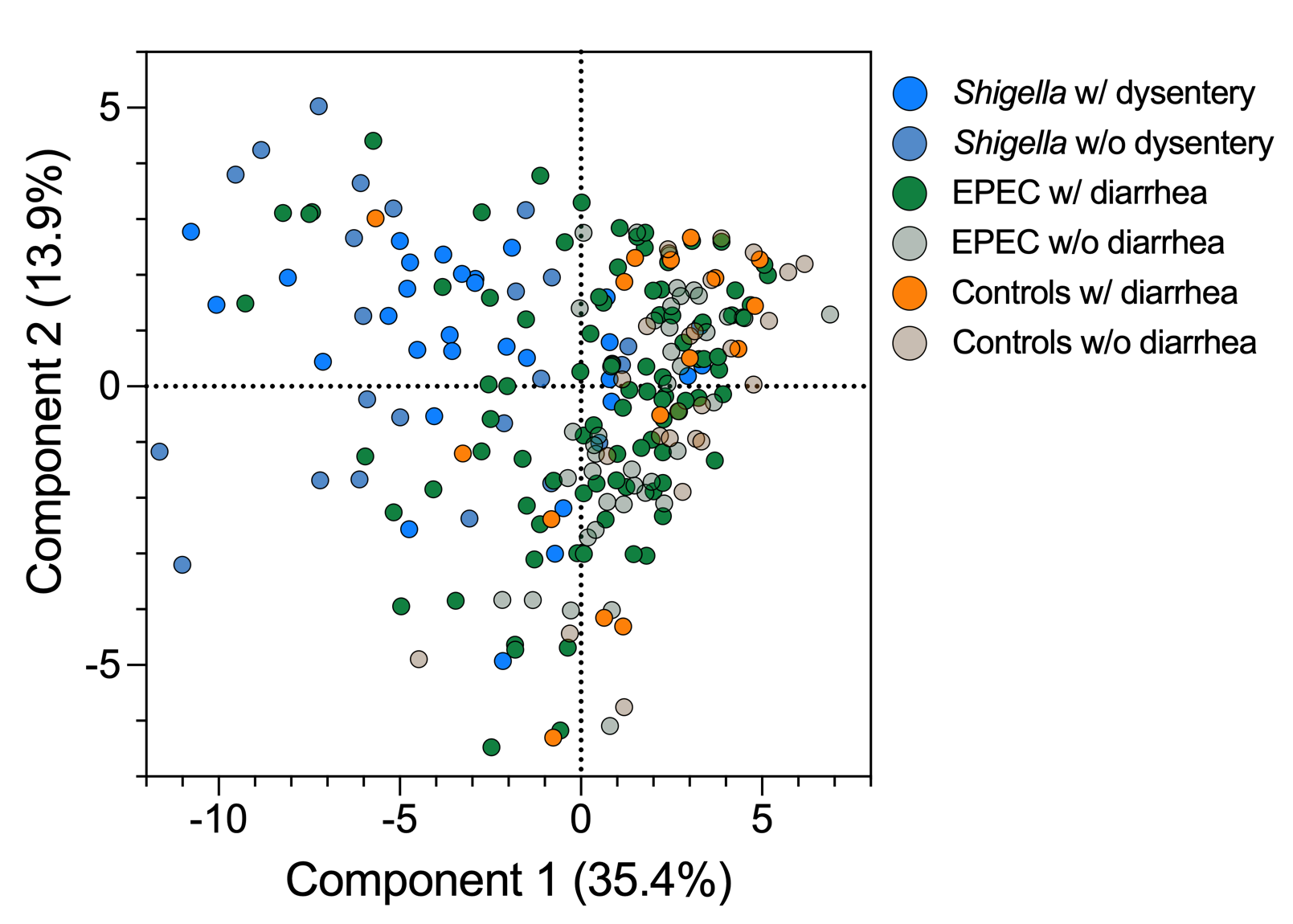

Supplement: FIG S1 [file mbio.00538-22-s0001.tif]

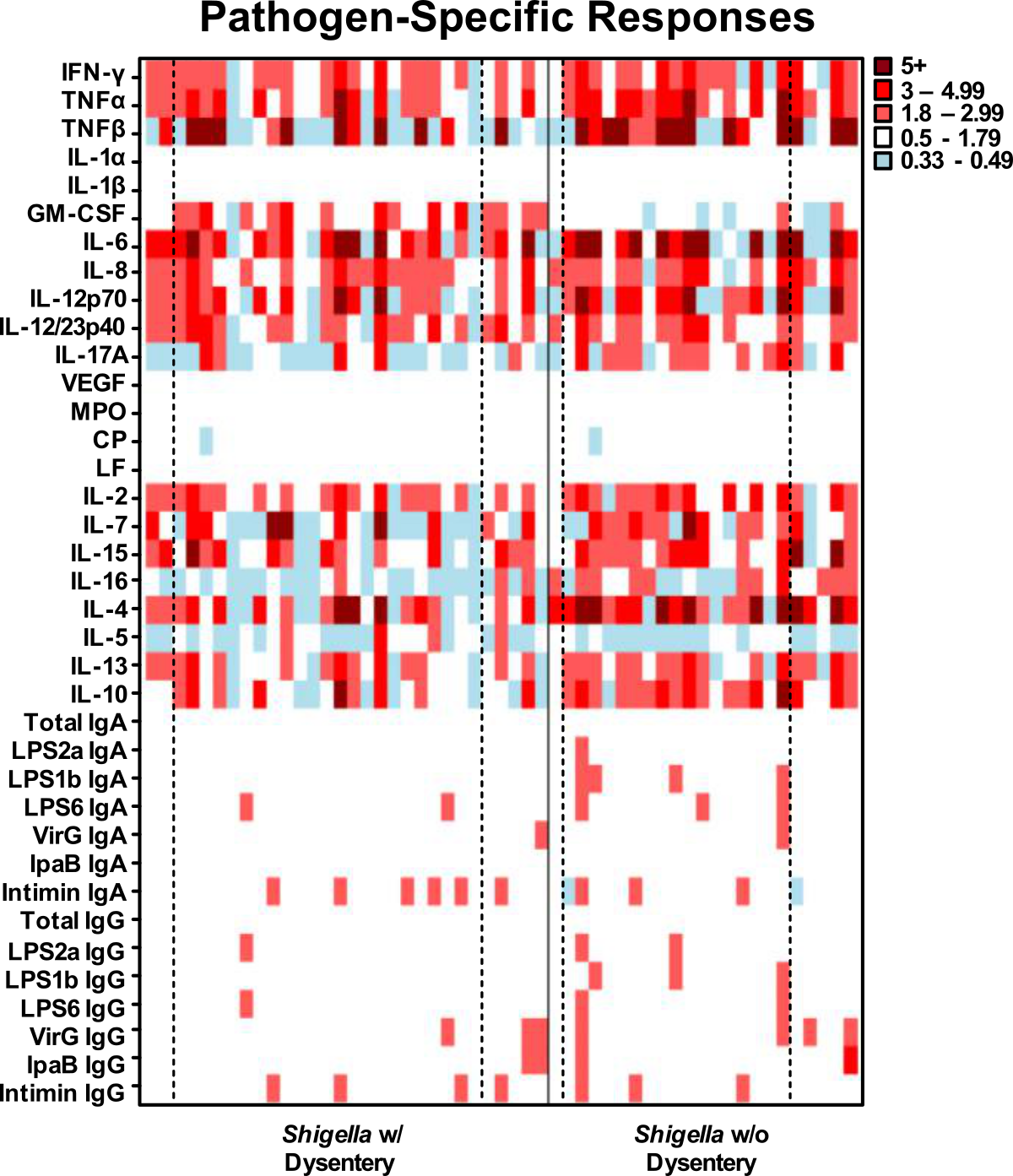

Supplement: FIG S2 [file mbio.00538-22-s0002.tif]

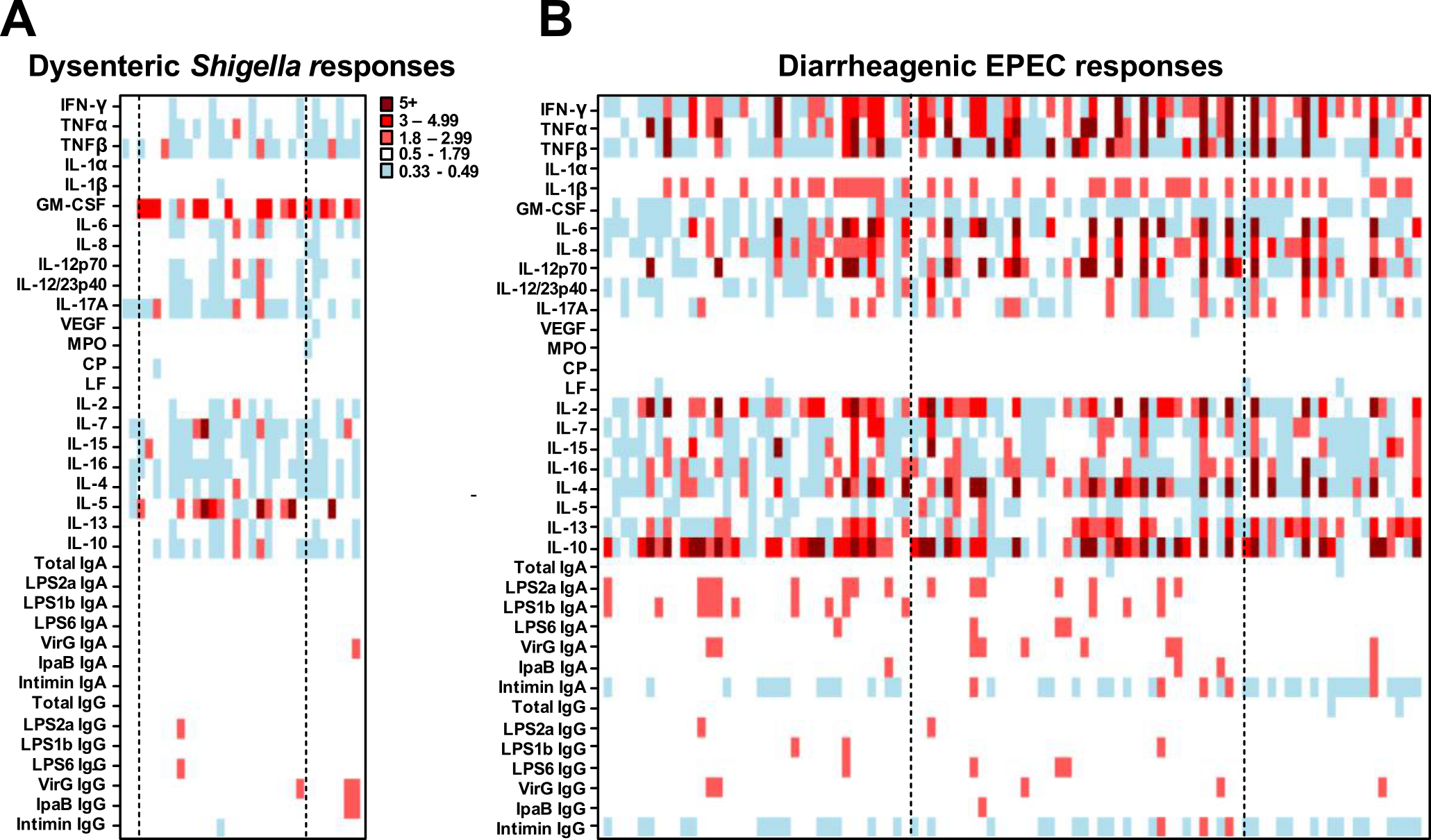

Supplement: FIG S3 [file mbio.00538-22-s0003.tif]

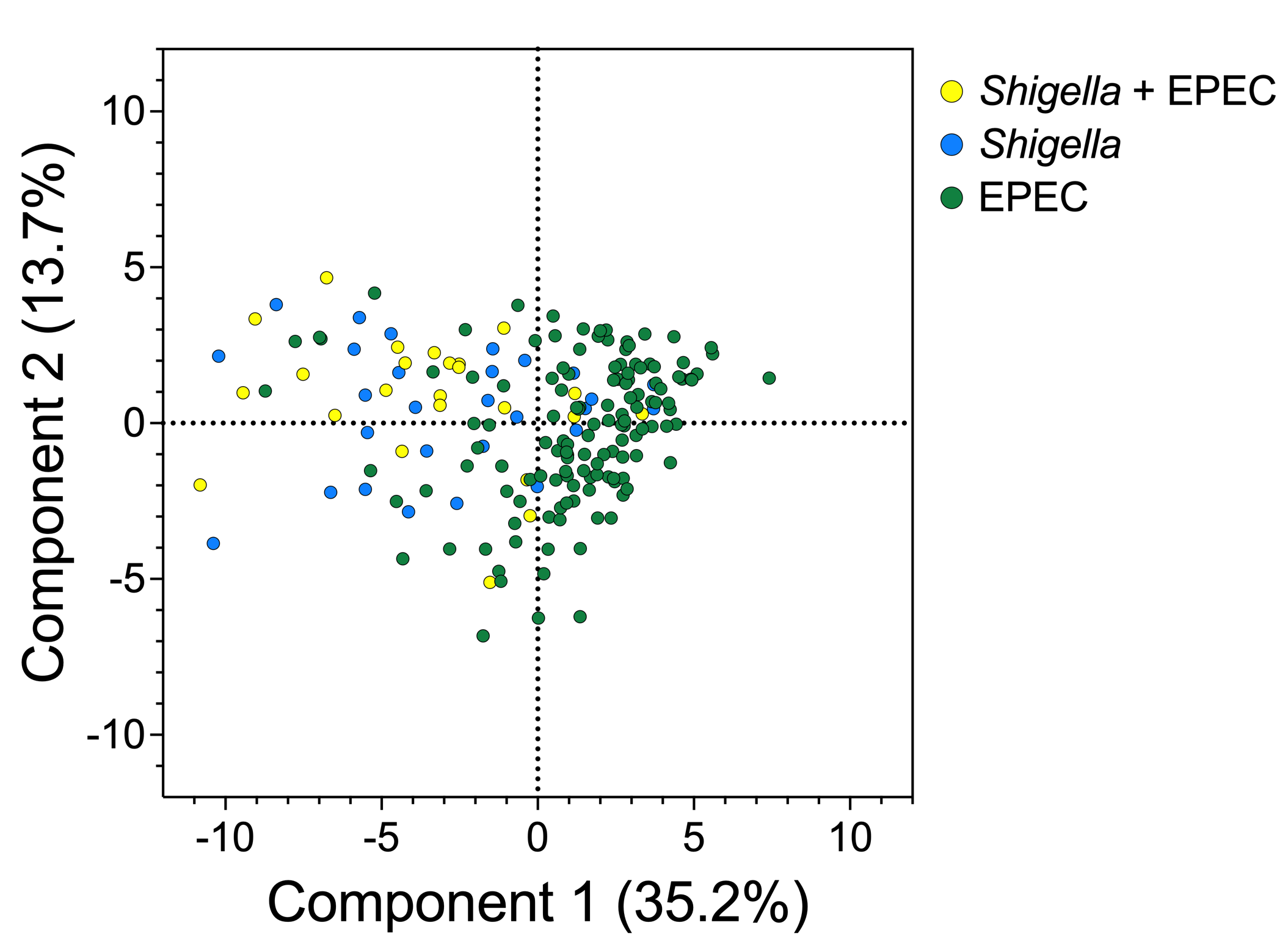

Supplement: FIG S4 [file mbio.00538-22-s0004.tif]

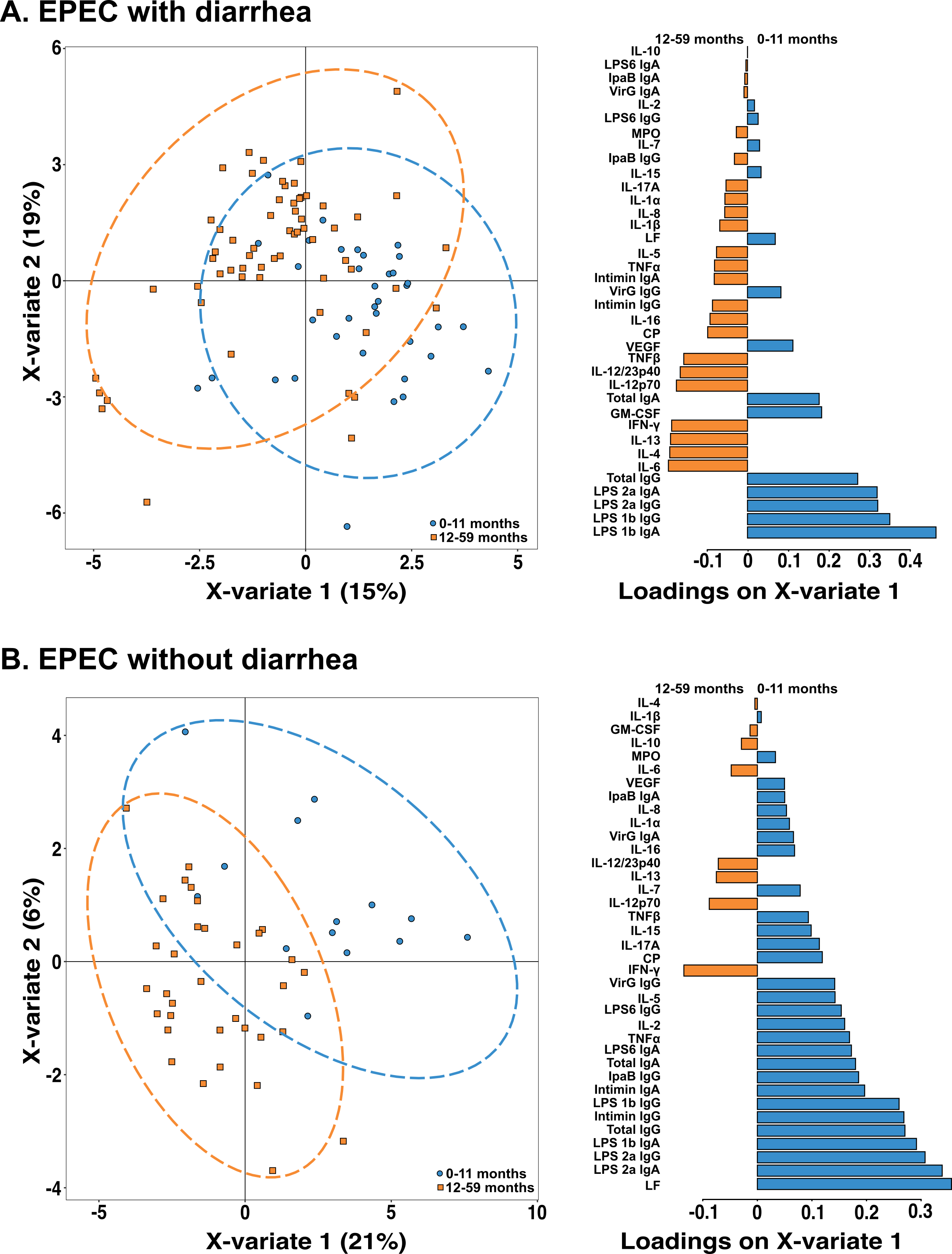

Supplement: FIG S5 [file mbio.00538-22-s0005.tif]
